# Comparison of photorefraction by Plusoptix A12 and cycloplegic autorefraction in children

**DOI:** 10.1186/s12886-024-03459-w

**Published:** 2024-04-19

**Authors:** Hadi Ghadimi, Mojgan Nikdel, Donny W. Suh

**Affiliations:** 1 Private Ophthalmology Practice, Atieh Medical Center, Rasht, 4144694198 Guilan Iran; 2 Department of Ophthalmology and Visual Science, Gavin Herbert Eye Institute, University of California at Irvine, Irvine, CA USA

**Keywords:** Amblyopia, Astigmatism, Cycloplegia, Hyperopia, Myopia, Refractive error

## Abstract

**Background:**

Plusoptix photoscreeners are capable of measuring refractive errors of children from 1 meter distance, without cyloplegia. We aimed to compare refractive data obtained from the newest version of Plusoptix (model 12) with cycloplegic autorefraction.

**Methods:**

We examined 111 consecutive children aged 3-7 years first by Plusoptix A12C under manifest condition and subsequently for cycloplegic refraction by Topcon KR-1 tabletop autorefractometer. Sphere, spherical equivalent, cylinder and axis of astigmatism measured by the two methods were analyzed to determine correlation, agreement and differences.

**Results:**

Binocular examination of 111 children aged 4.86±1.27 years revealed good agreement between refractive data obtained by Plusoptix and cycloautorefraction, according to Bland-Altman plots. Significant (*p* < 0.001) and strong correlation was found between all refractive measurements (Pearson’s r value of 0.707 for sphere, 0.756 for pherical equivalent, and 0.863 for cylinder). Plusoptix mean sphere, spherical equivalent and cylinder were 1.22, 0.56, and -1.32 D, respectively. Corresponding values for cycloautorefraction were 1.63, 1.00, and -1.26 D. The difference between axis of cylinder measured by the two methods was < 10° in 144 eyes (64.9%).

**Conclusions:**

Considering the significant agreement and correlation between Plusoptix photoscreener and cycloplegic autorefraction, the need for cycloplegic drops in refractive examination of children may be obviated. The mean difference between cylinder measurements are considerably trivial (0.06 D), but sphere is approximately 0.4 D underestimated by Plusoptix compared to cycloautorefraction, on average.

## Background

Recent population-based studies have estimated that 6.8% of children suffer from eye and vision conditions, with refractive errors being the most common disorder [1]. Undetected and untreated refractive errors not only affect the function and academic performance of the children, but may also potentially lead to amblyopia, a leading cause of vision impairment among children and young adults. A meta-analysis of 60 studies has shown a 2.4-2.9% prevalence of amblyopia in North America and Europe [2]. Timely detection and correction of refractive errors, the most common risk factor for amblyopia, have certainly proven to be effective in reduction of the prevalence and severity of amblyopia [3]. The best means of detection of amblyogenic risk factors is comprehensive pediatric ophthalmologist examination, including cycloplegic refraction [4]. However, many children are not comfortable with instillation of the cycloplegic eye drops and the consequent examination is time consuming and requires a still and cooperative child for achieving the most accurate results.

The Plusoptix photoscreeners have recently gained much popularity among physicians and patients as newer portable devices that detect refractive errors of both eyes simultaneously and without the need for cycloplegia. Unlike table-mounted or hand-held autorefractometers that require the patient to be examined from a very close distance, Plusoptix examination is considerably less threatening for the children since it is performed in less than 1 second and from a 1 meter distance. Prior studies have found the sensitivity and specificity of the newest generation of Plusoptix (model 12) to be >80% compared to the gold standard cycloplegic refraction amongst children [4–7]. In this study, we examined and compared the refractive measurements of children between 3-7 years using the Plusoptix A12 with cycloplegic autorefraction data.

## Materials and methods

In this prospective, observational study that was conducted in a private pediatric ophthalmology clinic between October 2021 and January 2022, consecutive children aged 3-7 years were included after providing written informed consent by their parents or legal guardians. The study and data collection were performed in accordance with relevant guidelines and regulations and adhered to local laws and the Institutional Review Board approval standards. The research protocol was approved by the ethics committee of Guilan society of ophthalmology and was compliant with the principles of the declaration of Helsinki. Exclusion criteria included the lack of cooperation for examinations, sensitivity to cycloplegic drops, various eye pathologies (poor ocular fixation or nystagmus, strabismus and media opacities like cataract or corneal haziness), and refractive errors exceeding the defined limits of Plusoptix (spherical equivalent refraction outside the range of -7.0 to +5.0 D).

Each patient was first examined by a trained nurse with Plusoptix A12C (Plusoptix GmbH, Nuremberg, Germany), prior to instillation of cycloplegic drops. The children sat on their parent’s lap for this examination and photorefraction of both eyes was performed simultaneously from 1 meter distance, where the nurse held the Plusoptix device. Subsequently, tropicamide 1% eye drops were instilled twice, separated by a 10 minute interval. Thirty minutes after the first drop, cycloplegic refraction was performed using tabletop Topcon KR-1 autorefractometer (Topcon Corporation, Tokyo, Japan). Cycloplegic examination and comprehensive ophthalmic evaluation were carried out by a pediatric ophthalmologist, blinded to the Plusoptix measurements. Refraction data of each patient were recorded as sphere, cylinder, and axis. All cylindrical values were recorded as negative cylinders and the spherical equivalent (SE) was calculated as sphere + cylinder/2. The differences for each parameter were calculated as the value measured by cyclorefraction minus the value measured by Plusoptix.

Statistical analysis was performed using IBM SPSS software version 25.0 (SPSS Inc, Chicago, IL). For comparison of the measurements made by the two devices, paired t-test and Pearson correlation were performed. The intraclass correlation coefficient was calculated and Bland-Altman graphs with 95% limits of agreement were also plotted to study the agreement between the two methods for refraction. P value less than 0.05 indicated statistical significance.

## Results

Both eyes of 111 children (54 females and 57 males) were included. Mean age was 4.86 ±1.27 years (range 3-7). Mean±SD of sphere, SE, cylinder and axis values measured by cyclo-autorefraction were 1.63±1.63 D, 1.00±1.69 D, -1.26±0.98 D, and 107.99±76.12° respectively. The corresponding measurements made by Plusoptix photorefractor were 1.22±1.38 D, 0.56±1.31 D, -1.32±0.99 D, and 89.13±74.6°. The p-values of paired t-test for differences between sphere, SE, cylinder and axis were < 0.001, <0.001, 0.106, and 0.001, respectively. In 144 eyes (64.9%), the difference between axis of cylinder measured by Plusoptix and cyclo-autorefraction was < 10°. In the remaining 35.1% of eyes, the mean±SD difference between cylinder axes of the two measurements was 29.6±22.1°.

**Fig. 1:**
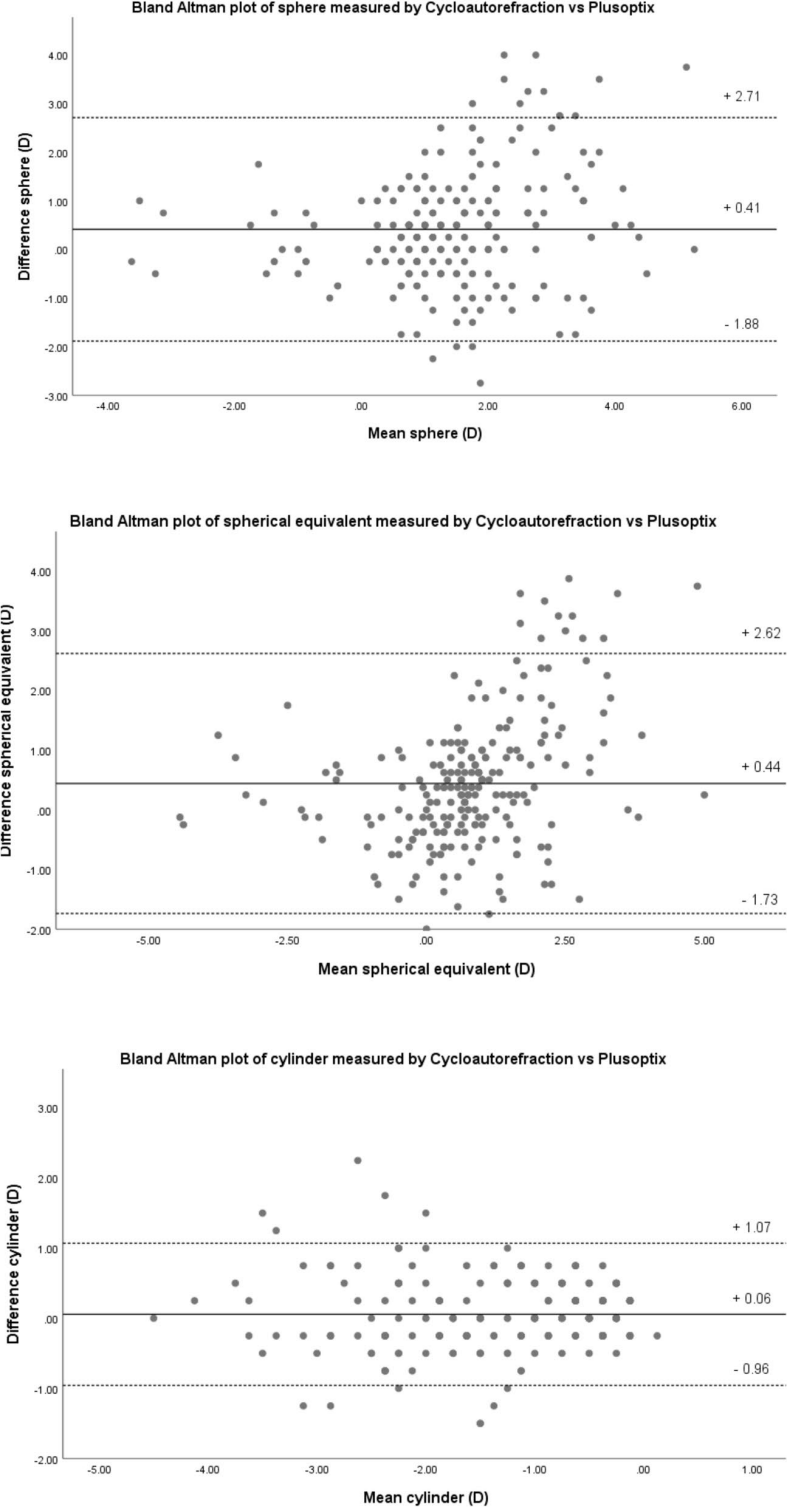
Bland-Altman plots illustrating agreement for sphere (top), spherical equivalent (middle), and cylinder (bottom). The difference between cycloautorefraction and Plusoptix measurements are plotted on the y-axis, and the mean values are plotted on the x-axis. Differences of the mean are shown by the solid line, and 95% upper and lower limits of agreement by the dotted lines

Correlation coefficient (r) between measurements by the two devices was 0.707 for sphere, 0.756 for SE, and 0.863 for cylinder. Corresponding intraclass coefficients (95% CI) were 0.805 (0.719-0.861),0.825 (0.734-0.880), and 0.926 (0.904-0.943). All correlation analyses were statistically significant (p < 0.001). The Bland-Altman plots for comparison of means and differences of refractive data measured by the two devices are shown in Fig. 1.

## Discussion

In 1974, Howland HC and Howland B introduced photorefraction as a technique to study refractive state of both eyes simultaneously from a 1-2 m distance [8]. In 1979, Kaakinen added photography of corneal light reflexes to photorefraction (recording fundus reflexes) to screen for strabismus as well as refractive errors which enabled clinicians to rule out major amblyogenic risk factors [9]. Plusoptix photoscreener has been introduced since 1995 [10]. The infrared light used in this instrument is advantageous because it is not perceived by the child as a light flash and is not threatening. The Plusoptix photoscreener has been approved by the US Food and Drug Administration (FDA) as a refractor [11], and provides non-cycloplegic refractive data including sphere, cylinder and axis. Moreover, significant strabismus (eye deviation >10°) or visual axis occlusion (eg, due to iris coloboma or blepharoptosis) are detected and alert messages displayed. The refractive measurements of the newest generation of Plusoptix, model 12, were compared in this study to the cycloplegic autorefractometer data.

We found a significantly high correlation between the measurements made by Plusoptix and cycloplegic autorefraction for all the refractive components. Although the sphere and spherical equivalent measured by Plusoptix were approximately 0.4 D less than cyclo-autorefraction on average, cylinder measurements were similar, with the mean difference being 0.06 D. Moreover, the difference in measurements of the axis of cylinder was less than 10° in approximately two-thirds of the eyes.

Refractive measurements by Plusoptix model 12 have been compared with cyclorefraction previously [12–17]. Some of these studies have used manual streak retinoscope for cyclorefraction and others have performed the cycloplegic exam by autorefractometers (table mounted devices like our study, or hand-held Retinomax autorefractor). Different age groups have also been included in the previous reports. Table 1 presents a comparison of the findings of previous publications to our results. Most of these studies revealed significant correlation and agreement between photorefraction and cyclorefraction. The mean differences between sphere, spherical equivalent, and cylinder measured by the two methods have ranged between 0.38-0.77, 0.27-1.51, and -0.17-0.37, respectively. Hence, our findings accord well with the results of the previous studies. All comparisons have showed very small difference between cylinder measurements, while sphere and SE determined by Plusoptix have been found to be partially underestimated.

**Table 1:** Findings of the studies comparing Plusoptix 12 and cycloplegic refraction of children

1st author, Year [Ref]	Cycloplegic Refraction	Plusoptix Model	Sample size	Age (yr) of participants	Mean diff Sphere (D)	Mean diff SE (D)	Mean diff Cylinder (D)	Mean diff Axis (°)
Current study	Tabletop autorefractometer Topcon KR-1	A12C	111 children, OU	3-7	0.41	0.44	0.06	18.9
Teberik, 2018 [12]	Tabletop autorefractometer Topcon KR-8100	A12	119 children, OU	6-17	0.29	0.38	-0.15	-10.8
Saini, 2019 [13]	Tabletop autorefractometer Potec PRK-6000	S12R	50 children, OU	8-15	0.69	0.74	0.10	13.5
	Manual retinoscopy	S12R	50 children, OU	3-7	0.68	0.77	0.18	39.4
Racano, 2019 [14]	Hand-held autorefractor Retinomax K-Plus 2	A12R	142 children, OU	3.2 ± 1.6	1.51	N/A	-0.17	N/A
Fogel-Levin, 2016 [15]	Manual retinoscopy	A12	201 children, OU	1-17	0.30	0.43	0.06	-6.0
Huang, 2017 [16]	Manual retinoscopy	A12C	357 children, OD only	3-4	0.27	0.45	0.37	N/A
AlHaddad, 2021 [17]	Manual retinoscopy	S12	37 children, OU	2-6	1.21	0.54	0.13	N/A

*Abbreviations: D* Diopter, diff: difference, *N/A* Not available, *OU* Both eyes, *OD* Right eye, *Ref* Reference, *SE* Spherical equivalent, *yr* year

Despite the statistical significance of differences of sphere measurements, the clinical significance of such discrepancies in refraction is considerably lower for the practitioners. Minor refractive errors do not need to be corrected. For high hyperopic refractive errors, partial hyperopic correction with 1.5 D less than full cyclorefraction is commonly practiced except in hyperopic strabismus [18]. Reduced hyperopic correction is more commonly practiced by both optometrists and ophthalmologists and even in cases of accommodative esotropia, partial correction is less likely to interfere with emmetropization compared to full correction of the hyperopia as determined by cycloplegic examination [19].

Interestingly, no study has compared Plusoptix model 12 with table mounted autorefractometers in the 3-7-years age group. To our knowledge, for the similar age group, there is only one paper comparing tabletop autorefractometer with an older model of Plusoptix (model 9). Yassa, et al. studied 70 eyes of 70 children aged 2-6 years and showed that mean sphere, SE and cylinder values measured by Plusoptix 9 were 1.16, 0.61, and -1.11 D, respectively. Corresponding data for cycloplegic examination performed using tabletop Nidek ARK-510A autorefractor were 1.60, 1.00, and -1.21 D [20]. Our findings with a newer version of Plusoptix photoscreener and a larger group of patients are very similar to their results.

Limitations to this study included exclusion of patients with high refractive errors that are beyond the range of measurements possible by Plusoptix photoscreener. In addition, cycloplegia was achieved using tropicamide eye drops, while many of the previous studies on Plusoptix model 12 have used cyclopentolate. Nevertheless, a meta-analysis showed that tropicamide could be considered as a viable alternative for cyclopentolate, because the difference in cyclorefraction after usage of these two drops was 0.17 D and not statistically significant [21]. In addition, tropicamide has been demonstrated to be strongly preferred over cyclopentolate by the patients [22]. Lastly, manual retinoscopy was not compared with the two other refractometers. Although cycloplegic retinoscopy with manual streak retinoscope is still widely considered as the historical gold standard for refraction in children, a meta-analysis revealed that cycloplegic autorefraction is an accurate and reliable alternative to cycloplegic retinoscopy, with difference in sphere or SE being < 0.25 D in most cases [23].

## Conclusions

This study demonstrates that Plusoptix A12 has acceptable agreement and strong correlation with cycloautorefraction in 3-7 year-old children. The mean difference is less than 0.5 D of sphere, and almost negligible in cylinder. Without the bothersome cycloplegic drop instillation that reduces a child’s compliance, photorefraction can present valuable refractive data that assist the practitioners in detection and appropriate prescription of spectacles for the children. Therefore, our findings provide further evidence in support of replacing cyclorefraction by non-cycloplegic Plusoptix photoscreening as a reliable alternative in children [24].

## Data Availability

Data are available on request only due to legal reasons. Requests should be directed to the corresponding author, Dr. Mojgan Nikdel, at: nikdel.mojgan@gmail.com. Address: Private ophthalmology practice, Atieh Medical Center, Rasht 4144694198, Iran. Tel/Fax: +98 13 33367073.
